# From COVID-19 to Green Recovery with natural capital accounting

**DOI:** 10.1007/s13280-022-01757-5

**Published:** 2022-07-27

**Authors:** Michael Vardon, Paul Lucas, Steve Bass, Matthew Agarwala, Andrea M. Bassi, Diane Coyle, Anthony Dvarskas, Catherine A. Farrell, Oliver Greenfield, Steven King, Martin Lok, Carl Obst, Brian O’Callaghan, Rosimeiry Portela, Juha Siikamäki

**Affiliations:** 1 Fenner School of Environment and Society, Australian National University, Canberra, ACT 0200 Australia; 2 PBL Netherlands Environmental Assessment Agency, P.O. Box 30314, 2500 GH Den Hague, The Netherlands; 3 IIED, 235 High Holborn, London, WC1V 7DN UK; 4 Alison Richard Building, 7 West Rd, Cambridge, CB3 9DT UK; 5 KnowlEdge Srl, via San Giovanni Battista 2, 21057 Olgiate Olona (VA), Italy; 6 320 Alison Richard Building, 7 West Rd, Cambridge, CB3 9DT UK; 7 PO Box 277, Kinderhook, NY 12106 USA; 8 EU LIFE On Machair Project, Westport, County Mayo Ireland; 9 235 High Holborn, London, WC1V 7DN UK; 10 219 Huntingdon Road, Cambridge, CB3 0DL UK; 11 Bezuidenhoutseweg 2, 2594 AV Den Haag, Netherlands; 12 219 Rathmines St, Fairfield, VIC 3078 Australia; 13 St John’s College, Oxford, OX1 3JP UK; 14 91 Phoenix Ave., Singapore, 668388 Singapore; 15 IUCN, 1630 Connecticut Avenue NW Suite 300, Washington, DC 20009 USA

**Keywords:** COVID-19, Green recovery, Natural capital accounting, Sustainable development, System of Environmental-Economic Accounting (SEEA)

## Abstract

The COVID-19 pandemic and related social and economic emergencies induced massive public spending and increased global debt. Economic recovery is now an opportunity to rebuild natural capital alongside financial, physical, social and human capital, for long-term societal benefit. Yet, current decision-making is dominated by economic imperatives and information systems that do not consider society’s dependence on natural capital and the ecosystem services it provides. New international standards for natural capital accounting (NCA) are now available to integrate environmental information into government decision-making. By revealing the effects of policies that influence natural capital, NCA supports identification, implementation and monitoring of Green Recovery pathways, including where environment and economy are most positively interlinked.

## Introduction

The COVID-19 pandemic has disrupted economies, societies and livelihoods around the globe. To mitigate health, unemployment and other socio-economic impacts, Governments quickly responded with increased resources for health care, followed by economic support packages for *economic rescue* to ease the impacts of national lock-downs and disrupted supply chains. More recently, Governments moved to *economic recovery* spending*,* aiming to restore employment and economic activity to pre-pandemic levels. This spending on economic rescue and recovery is of an unprecedented size and type, with unclear implications for sustainable development (O’Callaghan and Murdock 2021). Depending on the measures taken and their implementation, spending can have either long-lasting positive or negative impacts on the environment (Hepburn et al. 2020; OECD 2020, Piaggio and Siikamäki 2021; Vivid Economics and F4B 2021) and the natural capital on which society and the economy depend (IPBES 2019; Dasgupta 2021; UNEP 2021).

Natural capital is a term commonly used and is defined by Bateman and Mace (2020) as “those renewable and non-renewable natural resources (such as air, water, soils, and energy), stocks of which can benefit people both directly (for example, by delivering clean air) and indirectly (for example, by underpinning the economy)”. Biodiversity (genes, species and ecosystems) are a part of natural resources although they are not separately identified in the examples of this definition.

**Fig. 1:**
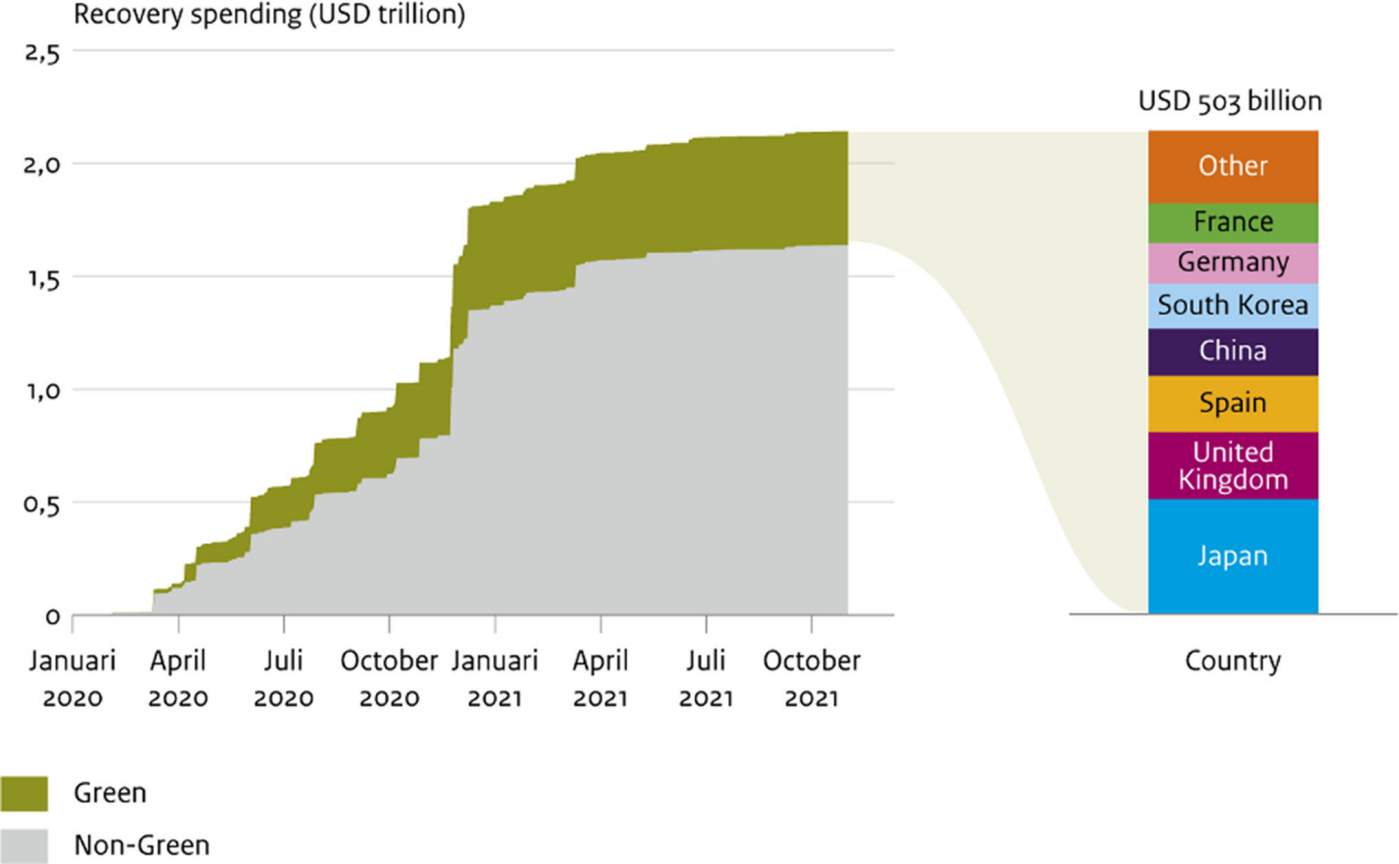
Green government recovery spending per November 2021. In this analysis, a “green” policy is one which advances any of the following priorities: climate mitigation, climate adaptation, natural capital, or air pollution reduction. *Source* Global Recovery Observatory  https://recovery.smithschool.ox.ac.uk/tracking/

Governments have announced increasing levels of recovery spending, totalling US$2.14 trillion in the first 18 months since April 2019 (Fig. 1). Most spending was by high-income countries. Dasgupta (2021) highlighted that investment in natural capital is an investment in the economy and society, but governments have not yet translated this understanding into economic recovery spending. Around 24% of announced recovery spending is ‘green’ (contributing to environmental objectives) and most is targeted at climate change mitigation, with 3% positive for natural capital, and up to 17% negative (O’Callaghan and Murdock 2021).

Nevertheless, there is increasing recognition that recovery efforts should not only address economic recovery, but should also be green, inclusive and resilient (Lucas and Vardon 2021). *Green*, in strengthening natural capital and addressing biodiversity loss and climate change; *inclusive*, in tackling the inequalities that the pandemic has exposed; and *resilient*, in preparing for future crises and shocks, including the impacts of climate change and biodiversity loss. Inadequate attention to the environmental dimensions of economic recovery spending makes achieving international goals, such as in the Paris Agreement^1^, the Post-2020 Global Biodiversity Framework^2^ and the UN Sustainable Development Goals (SDGs)^3^ harder to reach.

Decisions are often made with insufficient information or consideration of environmental pressures (e.g. by CO_2_ emissions, and waste, overexploitation of fish and forests) and dependencies on natural capital. This overlooks societal dependence on natural capital and economic and social gains from investments in natural capital (Dasgupta 2021; Piaggio and Siikamäki 2021; UNEP 2021). Natural Capital Accounting (NCA) provides coherent environmental and economic data that can be used in existing policy analysis, models and tools (Vardon et al. 2016; Bassi 2021; World Bank 2021b). NCA serves as a bridge between economists and environmental scientists, enabling better decision-making for sustainable development, by including information on the impacts and dependencies of society on natural capital (Ruijs et al. 2019).

The aim of this perspective is to demonstrate how NCA can help design, implement and monitor a Green Recovery and put the world on a sustainable development pathway. While business has a role in Green Recovery, and has adopted various forms of sustainability reporting (e.g. International Integrated Reporting Framework^4^ and Natural Capital Protocol^5^), the focus of this paper is on public policy and how to improve current national information systems that do not properly account for natural capital, hindering decision-making for sustainable development. It is recognised that the COVID-19 pandemic has had impacts on the environment (e.g. Rume and Islam 2020) but an examination of this is beyond the scope of this paper.

## Green Recovery and natural capital approaches

Economic recovery spending aims to increase aggregate demand and employment through direct capital investments, as well as expansionary fiscal and monetary policies and targeted sectoral policies, with high economy-wide spill-over effects (Lucas and Vardon 2021). By bringing human and environmental challenges, such as climate change and biodiversity loss, to the forefront, Green Recovery can combine short-term socio-economic recovery with medium- to long-term transitions to address persistent human and environmental challenges. Green Recovery builds on notions of green growth^6^, green economy^7^, circular economy (Stahel 2016) and sustainable development (WCED 1987), notions which have been broadly harmonised in the five principles for “inclusive green economies” facilitated by the Green Economy Coalition (Partners for Inclusive Green Economy, undated).

Three Green Recovery strategies can be distinguished, each with different ways of linking socio-economic recovery to the achievement of societal goals, including the Paris Agreement and the SDGs, and related sustainability transitions (Maas and Lucas 2021; Table 1). They describe a continuum of “shades of green”, ranging from incremental improvement to structural reform of the economy (Hopkins and Greenfield 2021) or transformative change of society (Díaz et al. 2019). Green Recovery initiatives are recent but growing; The Green Recovery Tracker^8^ reports on 41 countries with Green Recovery policies and shows more countries are developing such initiatives.

**Table 1:** Three strategies for green recovery

	Green as a co-benefit of recovery stimulus	Green as a necessary condition of recovery stimulus	Green as an opportunity for structural reform with recovery stimulus
Strategy	Measures for economic recovery also contribute to environmental goals and/or sustainable development	Conditionalities or safeguards are put in place to avoid investments and policies that increase environmental pressure or create stranded assets. This strategy thus excludes investments in environmentally harmful infrastructure (e.g. coal fired power plants)	Recovery measures are designed to make additional progress in the field of environmental goals and/or sustainable development. Green investments and policies are combined with structural reform, such as removing environmentally harmful subsidies or phasing out unsustainable practices
Recovery vs transition focus	Focus on socio-economic recovery. There is no direct coupling with long-term transitions	Focus on socio-economic recovery, while ensuring that this does not impede with long-term transitions	Socio-economic recovery goes hand in hand with long-term transitions
Natural capital focus	Recovery can also improve natural capital and its services	Recovery should not result in degradation of natural capital and its services	Recovery should improve natural capital and its services

*Source* Maas and Lucas (2021)

Despite some differences, each Green Recovery strategy is concerned with maintaining or enhancing natural capital for the benefit of current and future generations. They recognise that protecting, sustainably managing and restoring natural capital provides short-term employment and boosts economic growth, while at the same time delivering social benefits (such as improving health and food security), improving ecosystem services (e.g. flood control and carbon sequestration), reducing physical risks (e.g. flooding and storm-related damage) and helping to prevent future pandemics (Cohen-Shacham et al. 2016; IPBES 2019; Seddon et al. 2019; WWF and ILO 2020; Dobson et al. 2020; Palomo et al. 2021).

**Fig. 2:**
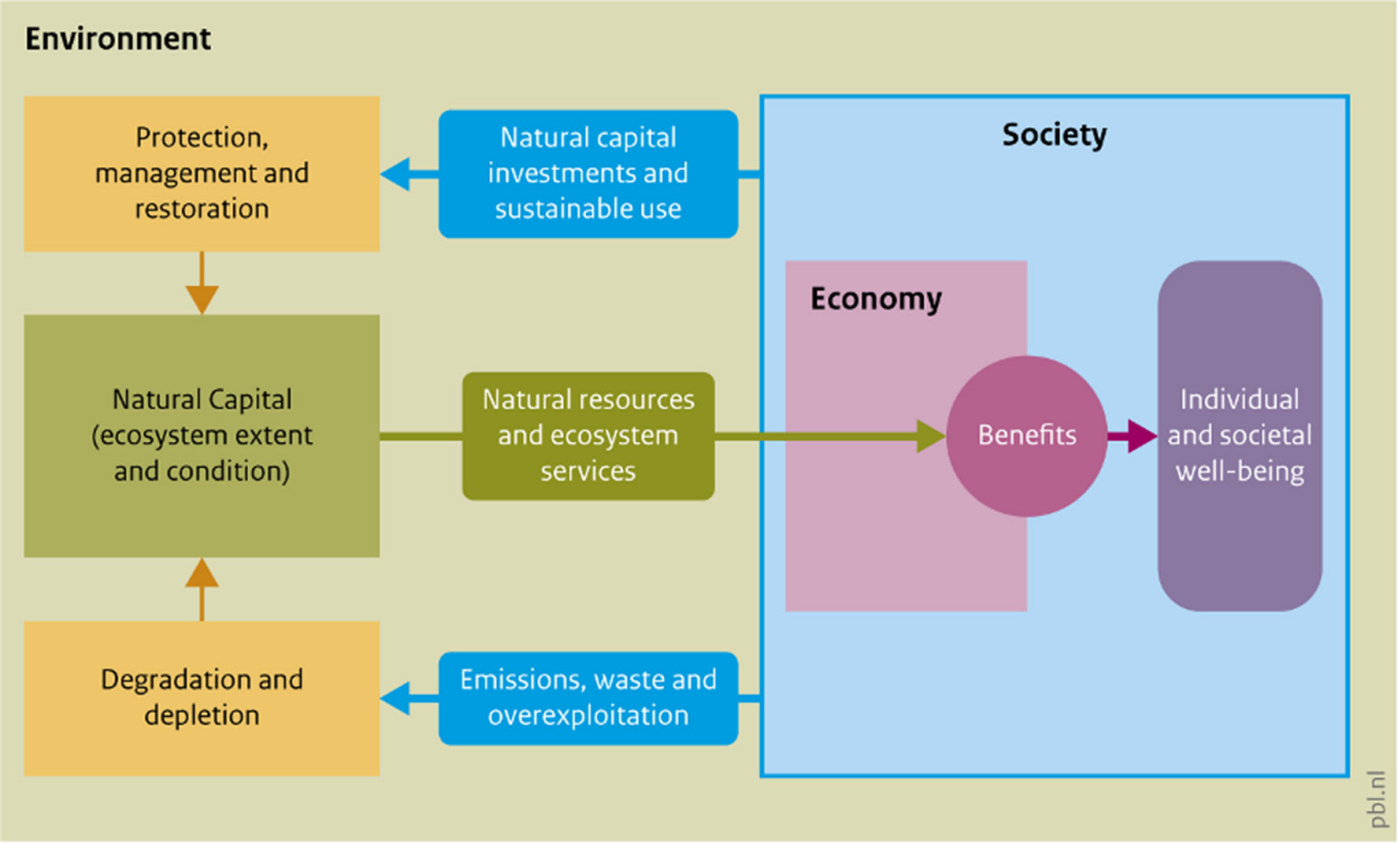
The interactions of the environment with society and the economy. *Source* Lucas and Vardon (2021)

Figure 2 is a conceptual model that shows how natural capital, society and the economy interact, and provides a framing for both Green Recovery and NCA. Spending on natural capital has two components: (1) enhancing natural capital through improved management and restoration to increase ecosystem extent and condition and the flow of ecosystem services, which is represented by the flow of investments from society for environmental protection and restoration at the top of Fig. 2, and (2) reducing environmental degradation and resource depletion through economic restructuring, as represented at the bottom of Fig. 2. Both components provide short-term economic benefits, while at the same time enhancing natural capital in the long term.

## Natural capital accounting

In response to the call in Agenda 21^9^ for the values of nature to be recognised in the information systems of governments, the System of Environmental-Economic Accounting (SEEA) was developed. The SEEA represents the global standard for NCA and is used by public information agencies like national statistical offices. The SEEA Central Framework (UN 2014) was adopted by the UN in 2012, and was followed by SEEA Ecosystem Accounting (UN 2021) in 2021. These frameworks integrate environmental data with the economic data from the System of National Accounts^10^ (SNA) that has played such a key role in decision-making. Among other things the SNA produces the widely used Gross Domestic Product (GDP) that is commonly misused as measure of progress (Coyle 2014). The integration of environmental and economic data serves to identify the *dependency* of people on the natural capital and ecosystem services they need for wellbeing and economic growth, and *the impact* of people’s activities on the environment (Fig. 2). The information from the SNA and the SEEA is arranged in a sequence of interlinked accounts (Table 2).

**Table 2:** Natural capital accounts relevant to Green Recovery

	System of national accounts (SNA) framework	System of environmental-economic accounting (SEEA) central framework	SEEA – Ecosystem asset accounts
Assets accounts	Economic asset accounts Change in economic assets on balance sheet items, financial capital, produced capital and non-produced capital (natural resources)	Environmental asset accounts Changes in stocks of e.g. minerals, energy sources, land, timber, aquatic resources, soil, water and biological resources	Ecosystem asset accounts^a^ Change in ecosystem extent (size), condition (quality) and capacity (future expected flows of ecosystem services)
Flow accounts	Economic supply and use tables Transactions by residents in the National Economy and income	Environmental supply and use tables Supply and use flows for energy, water, materials, incl. waste and emissions to soils, air and water Environmental protection activity account Transactions to preserve or protect the environment or to influence behaviour	Ecosystem supply and use tables Supply and use of intermediate and final ecosystem services flows (provisioning, regulating and cultural services)

*Source* Lucas and Vardon (2021)

From left to right, the three frameworks progressively include more aspects of the environment, mirroring the “shades of green”

^a^Including thematic accounts for climate change, biodiversity, oceans and urban areas

Having integrated and harmonised environmental and economic data in regularly updated accounts enables decision-makers to move beyond traditional siloed measures of economic success, notably GDP, which is based on obsolete economic theory and a mid-twentieth Century world-view that barely considered the environment (Stiglitz et al. 2010; Coyle 2014; Hamilton and Hepburn 2017; Dasgupta 2021). With NCA, which is based on an expansion of economic theory that recognises the importance of the environment, spending packages can be designed, tested, implemented, monitored and modified to achieve progress beyond GDP growth. NCA enables a course towards a more sustainable society to be charted, staying within ecological thresholds (Vardon et al. 2021) through a process of developing and using the accounts that creates a dialogue between different actors in society, thereby improving understanding, trust and vision (World Bank 2021b; Farrell et al. 2022).

NCA and the SEEA is not just theory. In 2020, 89 countries reported implementing the SEEA Central Framework, and 36 reported SEEA Ecosystem Accounting (UNCEEA 2021). The number of implementing countries reflects the substantial support provided by the international community to low- and middle-income countries channelled through programs like WAVES (Wealth Accounting and valuation of Ecosystem Services^11^) and NCAVES (NCA and Valuation of Ecosystem Services^12^). With growing exper-tise, experience, access to online data and modelling platforms (e.g. ARIES for SEEA^13^, EO4EA^14^ and InVEST^15^), it is increasingly possible to rapidly produce basic accounts (Lucas and Vardon 2021; World Bank 2021b).

Accounting is one part of an information system that supports decision-making. The other parts include basic data, analysis and modelling (Vardon et al. 2016). Accounting describes past trends and interactions, whereas policymaking requires looking forward and assessing present and future policy options (Bassi 2021). Modelling draws on the information from NCA to explore possible futures and policy applications. NCA and modelling have been combined in various ways to assess future impacts of alternative development pathways (Bassi 2021; Johnson et al. 2021). Several examples of using models with NCA make the case for investment in natural capital and ecosystem services (Collste et al. 2017; World Bank 2021c). While the use of models for examining environmental or economic issues is not new, the availability of integrated environmental and economic data from NCA makes it easier to feed models and analyse the interrelationships between the economy and the environment (Banerjee et al. 2020; Lucas and Vardon 2021).

## A Green Recovery through natural capital accounting

By providing integrated economic and environmental data NCA can improve decision-making for a Green Recovery. It brings together often disparate actors, supporting the alignment of multiple Green Recovery perspectives, shaping recovery packages so they build or improve natural capital and creating incentives for actors to cooperate (World Bank 2021b) with a compelling example from Ireland (Farrell et al. 2022). NCA also provides the impetus for improving the governance of natural capital, which too often incentivises degradation of natural (Vardon et al. 2021).

**Fig. 3:**
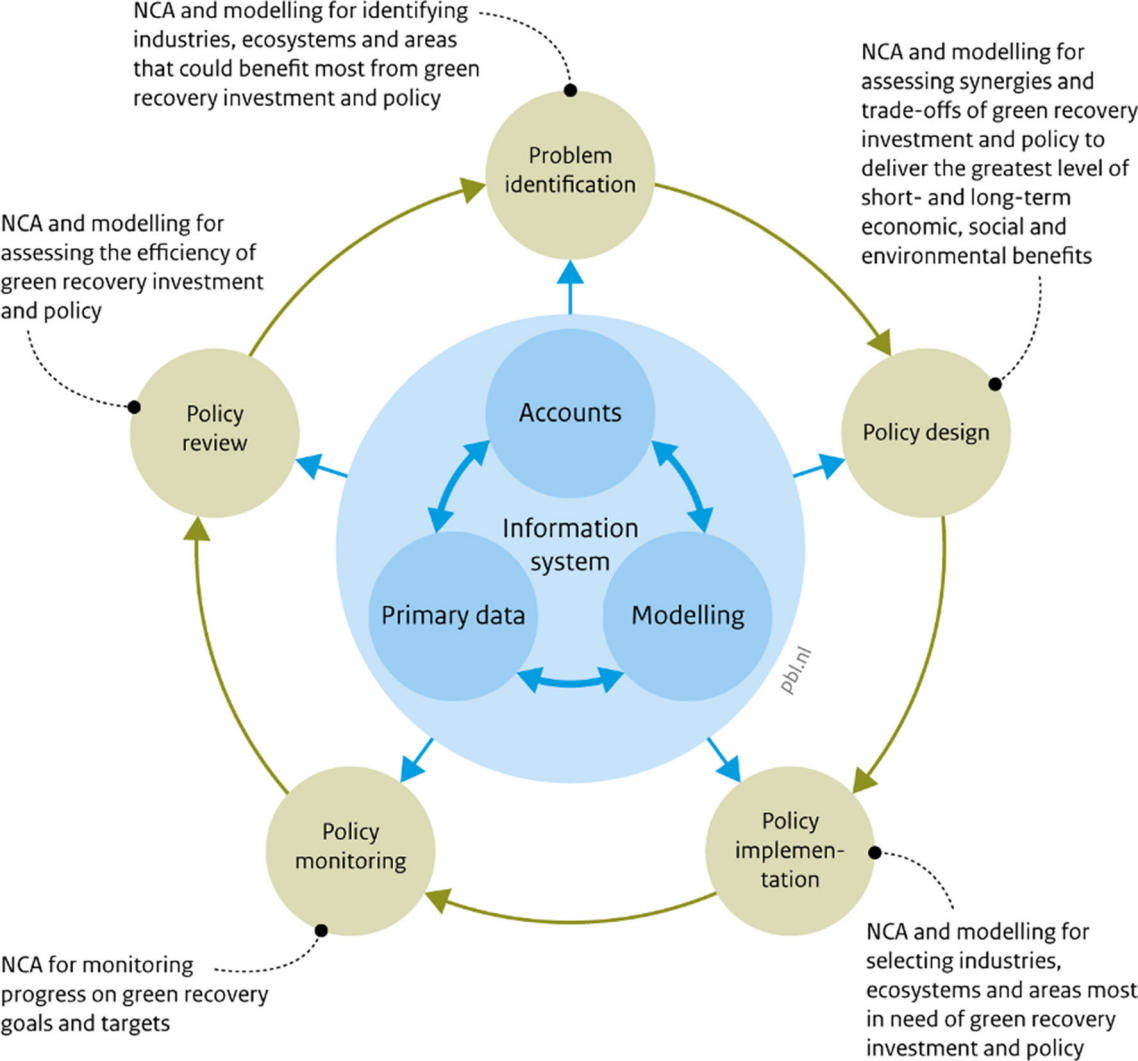
Use of NCA for Green Recovery across the policy cycle *Source* After Vardon et al. (2016)

NCA has been used by several countries for analysing issues aligned with Green Recovery, including biodiversity conservation and restoration (Coates et al. 2020; King et al. 2021; Farrell et al. 2022), tackling climate change (Pizarro 2020), integrated land management (Meijer et al. 2020) and SDG monitoring (Ruijs et al. 2018), with many examples from across the world (Vardon et al. 2017, 2019; Vardon and Bass 2020). Both the theoretical scope and practical examples reveal that NCA can be used in all phases of the typical policy cycle related to Green Recovery (Fig. 3; Table 3).

**Table 3:** Linking use of NCA to policy and questions of decision-makers

Policy uses	Decision makers’ questions	What information helps (data, accounts and analytical tools)	Types of answers that NCA can provide
Problem identification	How are we doing? What has changed, and how does that link to changes in the economy and other factors? Given assumptions about domestic and international development, how will we fare in the future?	Accounting data and derived indicators, simple projections, input–output analysis, environmental-economic models, scenario modelling, spatial analysis, footprint analysis	Interpretations from the data on past and present state Scenarios for future development of economy and environment
Policy design	If we want to change the current state or projected future state, what can we do? Who benefits from changes in policy? Who bears the costs of producing these benefits?	Accounting data and derived indicators, input–output analysis, computable general equilibrium modelling, environmental-economic models, scenario modelling, cost–benefit analysis, integrated assessment	Economic and environmental effects of restrictions on scenarios to achieve policy targets Ex ante assessment of the policies’ effects on the economy and environment
Policy implementation	How can we target the policy response to get the most improvement for the least cost? Which activities should be done first? What price should be put on natural resources?	Accounting data, derived indicators, environmental-economic modelling, spatial analysis, industry analysis, cost–benefit analysis, business case	Detailed assessment of all the pros and cons of the policy interventions
Policy monitoring	Are the policies making progress towards goals and targets?	Accounting data and derived indicators	Ex-post assessment of policy progress and evaluation of the need to adjust policy instruments
Policy review	How can we make the existing policy more effective to achieve the goals and targets? Are there any unintended consequences of the policy response? Do we need different policy responses?	Accounting data and derived indicators, econometric modelling	Ex-post policy evaluation of effectiveness and efficiency of policy instruments

*Source* Lucas and Vardon (2021)

Illustrations of how NCA can support policy and management in the various parts of the policy cycle are presented below for biodiversity conservation; climate action; SDG achievement; and finance and macroeconomic policy.

## NCA informing biodiversity conservation

**Fig. 4:**
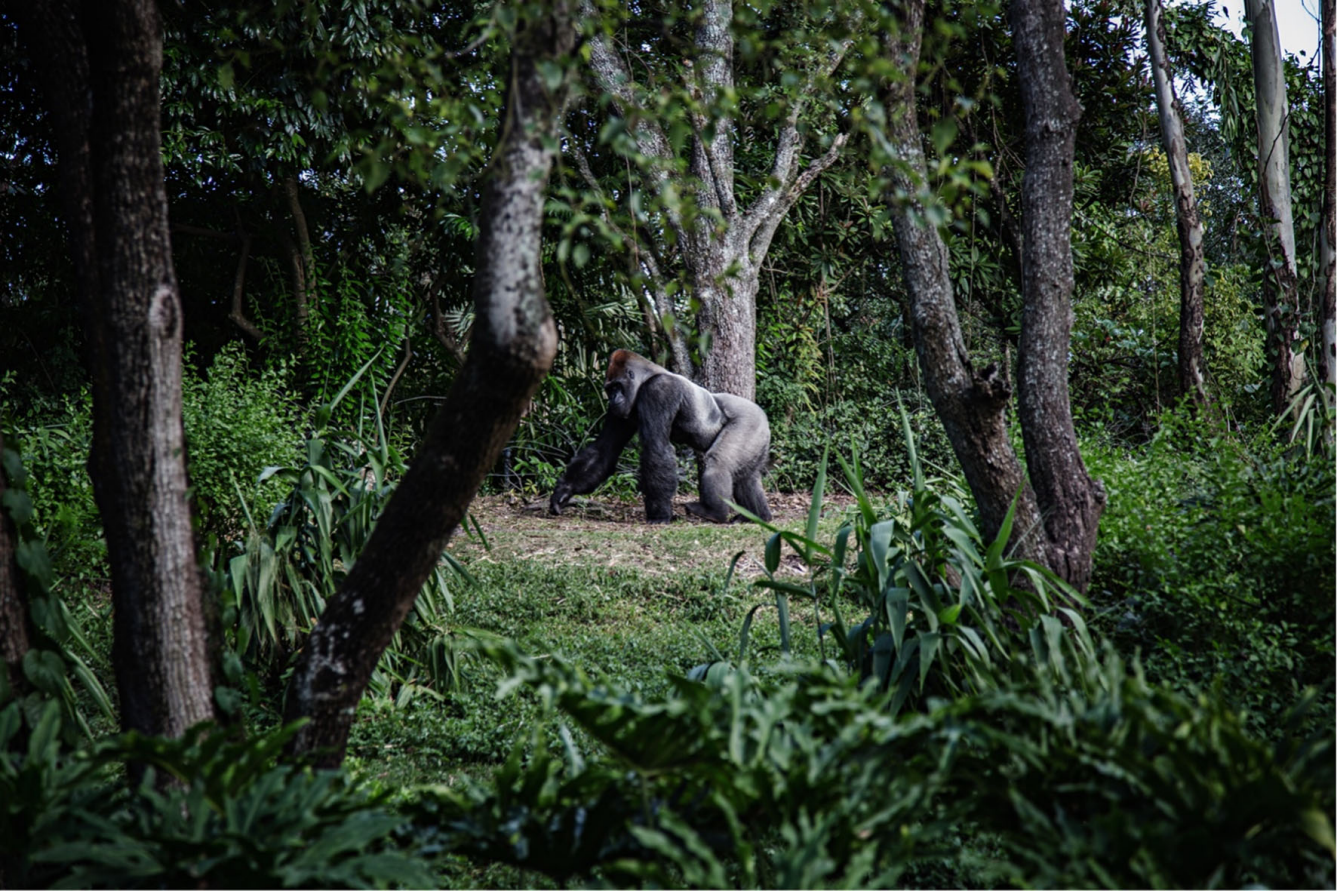
Iconic species like gorillas and the forests they inhabit can be managed so that they achieve social, economic and environmental benefits. Rwanda’s economic plan includes nature-based tourism and used evidence from natural capital accounts to estimate the level of investment needed to maintain the environment and restore tourism to pre-COVID-19 levels. Photo credit by Mike Arny and unplash https://unsplash.com/

Linking biodiversity indicators with national economic accounts provides a means of mainstreaming biodiversity policy into economic planning (Vardon et al. 2019; King et al. 2021). For example, NCA has been used to integrate economic development with biodiversity conservation in Rwanda, through the means of nature-based tourism. In 2019, tourism services were by far the largest source of foreign income for Rwanda, with much of this tourism related to iconic species, such as the Gorilla (*Gorilla beringei*) (Benitez et al. 2021). Survival of this iconic species, hence the tourism industry, is reliant on conservation measures (Fig. 4). In 2020, the COVID-19 pandemic led to a collapse of tourism in Rwanda, as it did all over Africa (African Union 2020). NCA played a prominent role in developing Rwanda’s recovery plan, providing the evidence needed to ensure the protection of ecosystems while demonstrating their role in economic development (Benitez et al. 2021). With the evidence from NCA, the Rwandan Government estimated an investment of US$3.9 billion was required to maintain the environment to ensure that nature-based tourism can return to pre-COVID-19 levels and continue to grow, while also providing ecosystem services like carbon sequestration and soil retention (Benitez et al. 2021). While the resources needed are not fully available, the accounts and the recovery plan provide a strong basis for seeking additional resources from development assistance agencies. Going forward NCA could also be used to monitor the effectiveness of expenditures in achieving environment and economic objectives, in this case the conservation of iconic species and employment and income from the tourism industry.

At a global level, the role of NCA is prominent in the first draft of the CBD Post-2020 Global Biodiversity Framework^16^, which recognises the importance of embedding the value of biodiversity in decision-making and promotes transparency with the implementation of a natural capital accounting framework. NCA can help to achieve Goal B “*Nature’s contributions to people are valued, maintained or enhanced through conservation and sustainable use supporting the global development agenda for the benefit of all*” (targets 14–16 and related indicators). This is also recognised in the CBD’s Action Plan for the Long-term Approach to Mainstreaming Biodiversity^17^ which identifies the need to “*develop and implement nature and biodiversity reporting and implement ecosystem or natural capital accounting, using the SEEA-framework as part of national accounts to inform decision-making and implementation*” (CBD 2020, proposed activity 1.1.3).

## NCA informing climate action

Natural capital investments provide benefits for climate change mitigation and adaptation (Klenert et al. 2020). For example, many ecosystems sequester and store carbon while some ecosystem types provide resilience against climate change (e.g. mangroves provide coastal protection from storm surges). The 2021 UN Climate Change Conference (COP26) in Glasgow attempted to bring the climate change and biodiversity agendas closer together. Its final outcome document, the Glasgow Climate Pact^18^, emphasised “the importance of protecting, conserving and restoring nature and ecosystems to achieve the Paris Agreement …” (paragraph 38). Specific ways to bring these agendas together were not identified but, as argued in this paper, information from NCA can support the decision-making process in both agendas and adopting NCA as an information source would thus be a starting point. This would help to identify synergies, for example where investment can create the greatest amount of benefits for least cost.

For climate action, accounts of greenhouse gas emissions and carbon can show progress towards achieving the aim of the Paris Agreement to hold the increase in the global average temperature to “well below” 2 °C above pre-industrial levels. Unlike UNFCCC reporting, SEEA-based accounts directly link emissions to the SNA (Keith et al. 2021). As such, NCA provides model-ready data to help countries assess the impacts on different sectors of transitioning to a low-carbon economy.

**Fig. 5:**
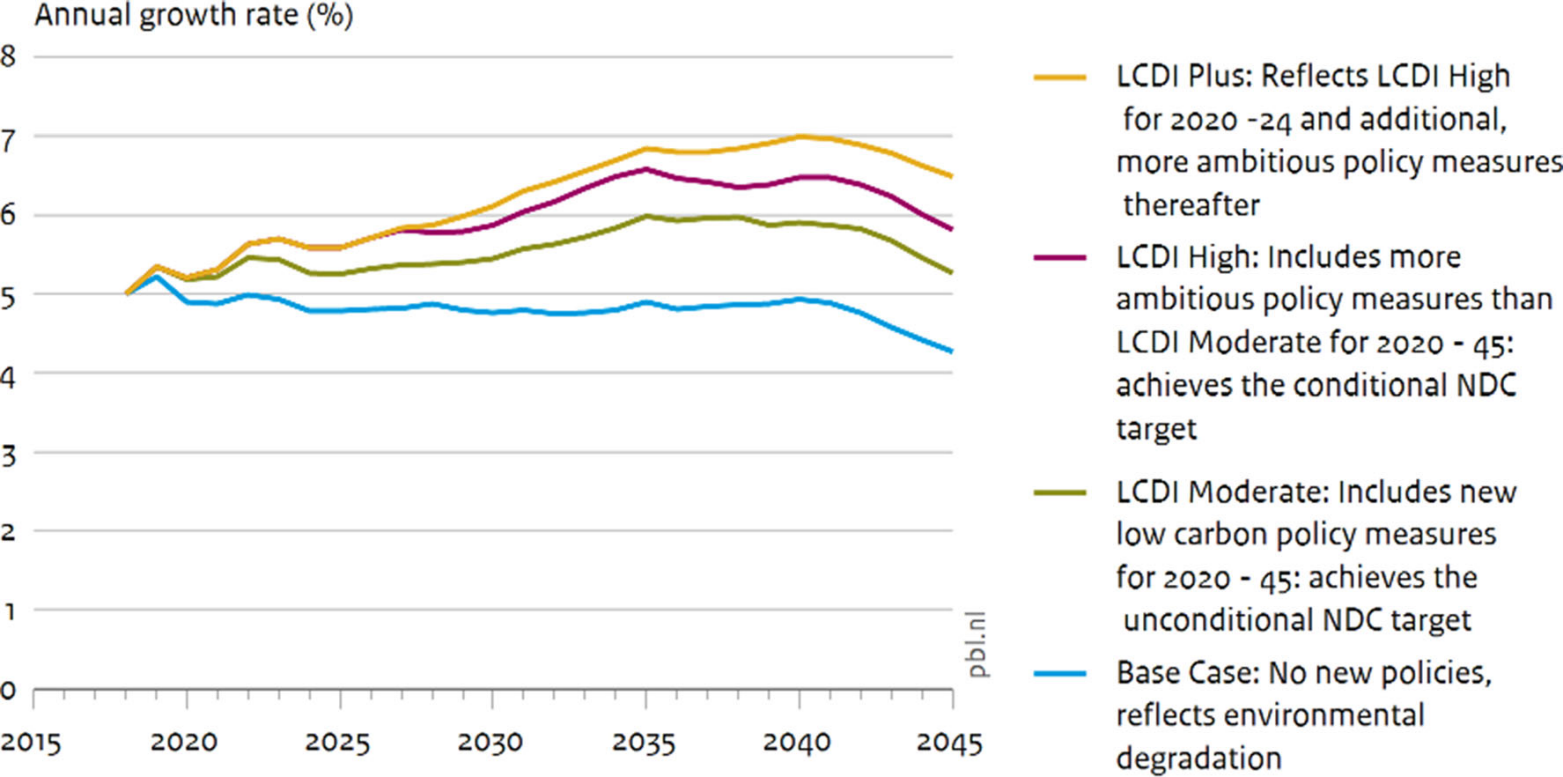
GDP and four growth scenarios by level of CO_2_ emissions in Indonesia. *Source* (BAPPENAS 2019)

For example, NCA informed the Government of Indonesia’s Medium Term Development Plan 2020–2024 (BAPPENAS 2019) with a Low Carbon Development Initiative (LCDI) assessing four different development pathways using scenario modelling. The LCDI scenarios examined the impacts of the medium-term strategy up to 2024, the Nationally Determined Contributions (NDCs) of Indonesia up to 2030, as well as further policy action beyond 2030, on several indicators including GDP growth, forest loss, jobs, air quality and poverty. The GDP projections account for the impact of environmental pressures, which grow under a baseline scenario and decline when climate mitigation and adaptation interventions are implemented. The analysis showed that a low-carbon growth path could deliver an average annual GDP growth rate of 6% (Fig. 5) while also unlocking an array of economic, social and environmental benefits, including reducing extreme poverty, generating additional better-paid employment and reducing mortality due to lower air pollution. These scenarios were updated in 2021 to support the preparation of a Green Recovery strategy (BAPPENAS 2021). This extended the ambition for low-carbon development to achieving Net Zero by 2060. The concept of environmental carrying capacity, embedded in the analysis with NCA, was central to the government’s analysis and post-COVID-19 recovery strategy.

Investments in green space also provide benefits to climate change. Heris et al. (2021) used NCA to assess the economic benefits of urban trees in US cities for two ecosystem services: (1) cooling and mitigating climate change, and thereby reducing the need for air conditioning, and (2) rainfall interception providing improved water quality and flood mitigation. The value of these two services for 768 US cities in 2016 was estimated at US$ 539 million and US$ 425 million, respectively. In up to 11% of these cities, investing in natural capital for climate mitigation and adaptation (green infrastructure) was determined to reduce costs (51% less), and to generate additional benefits (28% more) when compared to equivalent “grey” infrastructure (produced capital) (Bassi et al. 2021). Outside of urban areas, investments to address climate change have other additional benefits. For example, the restoration or conservation of forested areas not only stores and sequesters carbon but also helps to achieve the goals of the Convention on Biological Diversity and provides other ecosystem services that support activities such as ecotourism that can help economies recover from COVID-19. Increased availability of NCA could make these types of assessment routine and guide recovery spending to those natural capital investments that lead to higher social and economic returns by working with nature.

## NCA informing achievement of the Sustainable Development Goals (SDGs)

In 2015, the world committed to the 17 Sustainable Development Goals^19^ (SDGs), to achieve a prosperous, socially inclusive and environmentally sustainable future for people and the planet. The SDGs are a universal agenda building on the Millennium Development Goals^20^. The SDGs are “integrated and indivisible and balance the three dimensions of sustainable development: the economic, social and environmental”^21^. As NCA is an integrated information system it can inform the design, implementation and review of evidence-based SDG policies.

Up to forty SDG indicators can be derived directly from the SEEA—notably SDG 6 (water), SDG 13 (climate), SDG 14 (life below water) and SDG 15 (life on land) (UN 2019). Examples from Rwanda, Botswana, Brazil, the Netherlands and Sweden show that accounts-based data could potentially be used to derive indicators for SDG 2 (agriculture), SDG 7 (energy), SDG 8 (employment and economic growth), SDG 9 (industry, innovation and infrastructure), SDG 11 (cities) and SDG 12 (sustainable consumption and production) (Ruijs et al. 2018). NCA also provides the information necessary to target and monitor the structural reforms needed for achieving the SDGs. Implementation of NCA is itself an indicator for SDG target 17.9 on capacity building and for SDG target 17.19 on supporting statistical capacity building in developing countries. Without this support, many low- and middle-income countries will not be able to develop and apply NCA.

Other countries are using NCA to help achieve particular SDGs. For example, in Colombia water accounts and modelling were used to assess catchment management costs to provide clean water to support basic human needs and economic production (SDG 6: clean water and sanitation) (Romero et al. 2017). In Australia, NCA was also used to show the relative value of water provisioning compared to timber provisioning and other economic activities, arguing for a cessation of logging native forest (Keith et al. 2017) and a plan to phase out logging^22^ was subsequently made by government.

## NCA informing finance and macroeconomic policy

NCA can contribute to sustainable finance and macroeconomic policy. Alongside produced, human and social capital, natural capital is a core component of national wealth (Managi and Kumar 2018; Mandle et al. 2019; Zenghelis et al. 2020; World Bank 2021a). As is the case for any capital asset, the relevant, reliable and timely measurement of natural capital is necessary for efficient management. Understanding trends in the quantity, quality and value of assets and the services they deliver helps identify investment priorities and aids the design of incentive mechanisms that support green and fair outcomes.

Specific policy objectives vary between countries, but governments generally focus on growth (for job creation and improving living standards) and financial stability. The “fiscal triangle” illustrates the day-to-day management of government finances, balancing taxation, borrowing and spending. Public spending on economic recovery is funded by current taxes and debt. Debt is serviced through future taxes.

**Fig. 6:**
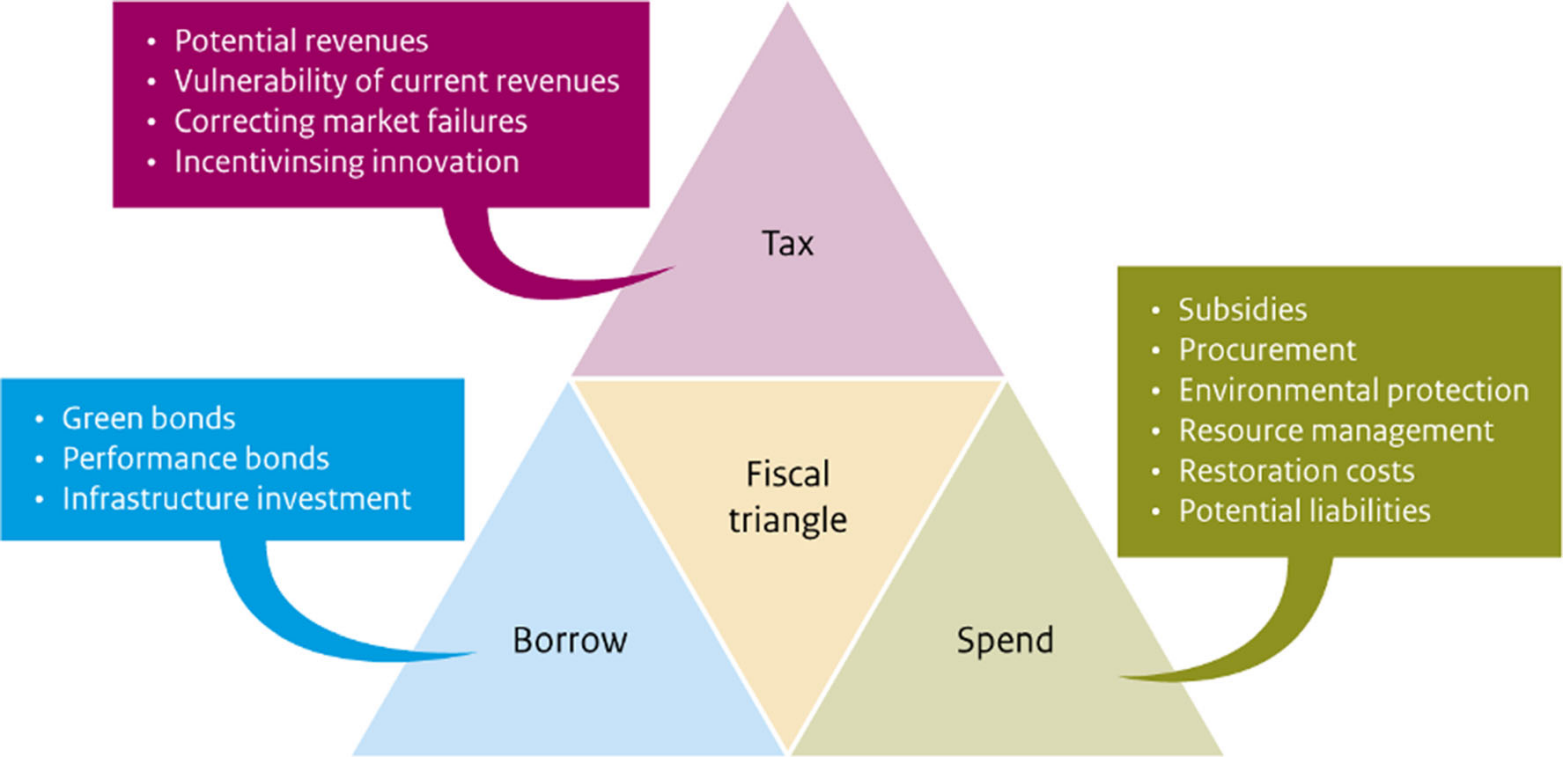
The fiscal triangle and examples of the relationship to NCA and policy. *Source* After Agarwala and Zenghelis (2020)

NCA relates to all parts of the fiscal triangle (Fig. 6). On the *expenditure* side, accounts record spending on environmental protection and restoration, resource management and subsidies (or foregone income) which may harm or benefit nature. Such expenditure, by both the public and private sector, may be considered investments in natural capital. A decline in natural capital represents the accumulation of debt, much of which is borne by the public sector and future generations (Dasgupta 2021; Vardon et al. 2021). The cost of restoration (offsetting the depreciation in the condition of natural capital or reducing the debt) may be calculated from the expenditures and subsidies recorded. Some natural capital debts, like species extinction, cannot be repaid.

For *taxation*, the accounts provide a perspective on the reliability of different revenue streams, such as carbon taxes compared to fuel duties. For instance, the UK’s natural capital accounts show that COVID-19 travel restrictions reduced fuel duty revenues by 19% from 2019 to 2020 (ONS 2021). The accounts demonstrate that fuel duty provides more than half of all revenue from environmental taxes, and that an alternative revenue stream is needed as drivers switch to electric vehicles. Environmentally harmful subsidies are not included in the UK’s accounts, but they are within scope NCA and in the past some countries have estimated the value of these for the energy and other industries (Palm and Larsson 2007). The accounts can also be used to identify links between the environment and other areas of taxation and spending. For example, natural capital investments that improve health (e.g. by improving air quality) can spur additional tax revenues due to increased labour productivity, while simultaneously reducing expenditure on treating respiratory illnesses. Harmful air pollutants (e.g. PM 2.5) from fossil fuels cause millions of deaths annually (Vohra et al. 2021) and removal of fossils fuel subsidies could also reduce the associated costs.

Finally, NCA can also play a role on the debt or *borrowing* side of the fiscal triangle. For example, green bonds are intended to finance environment-friendly investments in low-carbon infrastructure, flood management, ecosystem restoration and biodiversity conservation. Green bonds are issued by sovereigns (governments) and corporations, with more than 8,000 already listed in the Nasdaq Sustainable Bond Network^23^. The Climate Bonds Initiative^24^ puts the value of issued green bonds in excess of USD $1.5 trillion, with the potential for more than US$1 trillion to be added in 2022. Natural capital accounts provide evidence for assessing if the investments financed by green bonds lead to the expected environmental benefits, helping reduce the risk of “greenwashing”. NCA can also guide the green bond market towards sectors that yield both economic and environmental returns. NCA can reveal declines in natural capital that increase the risk of climate change, which could to lead to the downgrading of sovereign credit in many countries—or it could demonstrate net natural capital gains (Klusak et al. 2021).

## Conclusions and next steps

The need for government to consider the environment in development planning and economic management has been recognised for decades (WCED 1987). NCA provides a way for environmental information to be integrated with mainstream economic information, in turn informing the modelling and analysis that governments use for development planning and economic management.

Following the COVID-19 pandemic, government recovery stimulus has been extensive, and has increased over time, but has not fully considered the environment. Further recovery stimulus must, at least, avoid harm by taking into account the impacts of spending on the environment and, at best, provide the resources and associated structural reforms needed to enhance natural capital’s role in providing future social, economic and environmental benefits.

As the examples presented in this paper show, NCA can assist the development and implementation of policies, programs and financing needed for Green Recovery. It provides comprehensive information on natural capital and society’s dependencies and impacts on it. Furthermore, it can assist monitoring the impact of spending on the economy and the environment and can foster an enabling environment for actors to cooperate and transform the way they take decisions. In doing so, NCA informs evidence-based and dynamic policies.

As such, NCA is a means for achieving a wide range of Green Recovery goals, whether they be healthy livelihoods, sustainable production and consumption, biodiversity conservation and restoration, lowering greenhouse gas emissions, or realisation of the SDGs. NCA highlights the linkages between these diverse agendas, helping to find synergies and avoid trade-offs.

Authorities in charge of recovery stimulus and reform instruments can use NCA across the policy cycle (from policy design, planning and financing to implementation and monitoring) to ‘reset’ the economy to deliver Green Recovery, simultaneously reinvigorating economic growth, arresting environmental decline and achieving a more equitable society. Indeed, all parts of government have a stake in Green Recovery and NCA can provide them with consistent and regularly updated information for planning, implementation and monitoring.

NCA will be more effective when mainstreamed in the government machinery and decision-centred in its content and delivery. The Policy Forum on Natural Capital Accounting for Better Decision Making^25^, established by the World Bank’s WAVES Partnership in 2017, aims to share, explore and synthesise the experiences of countries that have been producing and using NCA. The Policy Forum convened five times and each time proceedings were published (World Bank 2021b). Emerging from the forum were ten principles for making NCA fit for policy (Ruijs et al. 2019), three of which relate to mainstreaming accounting: enduring NCA, continuously improving NCA and embedding NCA in government. Having on-going accounts means that a regular and increasing amount of data are available for analysis. This allows for continuously improving accounts, taking advantage of new and evolving data sources, and assessing changes over time. Embedding NCA into government decision-making processes—and relevant private sector and civil society processes too—can improve data harmonisation, reliability and transparency as well as reduce data duplication and access costs. Over time, understanding and trust of NCA and the decisions it informs should increase.

While NCA is a potential catalyst for Green Recovery, a critical next step is to ensure purposeful action, which will require investment in information. Although accounts are proliferating and some underpinning data are improving over time, account production is still dependent on basic data collection and appropriate expertise. New data sources and online platforms can help, and the growing capacity to produce and use NCA can be better mobilised. Basic accounts can now be developed with relative ease. But support is required to continue to strengthen the capacity of all countries, and particularly low- and middle-income countries, to produce NCA and ensure that they are relevant to, and hence can influence, government decision-making and policy.

At present NCA and Green Recovery awareness is limited. Without an understanding of the fundamental features of both, uptake of either will be hindered and ‘nature-negative’ GDP growth will likely prevail. Better communication is needed of what Green Recovery and NCA are (Tables 1 and 2, respectively) and how they can benefit decision-making in general (Fig. 3), with specific examples, such as those presented and referred to in this paper, used to demonstrate it is more than theory. To increase awareness, the NCA and Green Recovery communities should continue collaborating, engaging with decision-makers to help them better understand, use and trust NCA so they can be applied to Green Recovery. Such engagement will help to gain the investment in NCA, and the institutional reforms needed for Green Recovery.

The imperative now is to turn the combined adoption of the new NCA standards and the unprecedented levels of government spending for economic recovery, to move along the sustainable development pathway. Many options are available to make such a Green Recovery a reality. To achieve this a multidisciplinary effort is needed with people and institutions working together to promote NCA so that it is embedded, trusted and used in decision-making. With NCA government agencies responsible for assessing, funding and implementing recovery programs will have the information needed to plan and implement Green Recovery. Without improved information governments will not have the opportunity to fully consider the environment in decision-making. If future spending decisions continue to largely ignore the environment, then future generations will be saddled with massive debts, less ability to repay these debts as a result of reduced natural capital and ultimately an unsustainable society.

## Footnotes

1 https://unfccc.int/sites/default/files/english_paris_agreement.pdf

2 https://www.cbd.int/doc/c/abb5/591f/2e46096d3f0330b08ce87a45/wg2020-03-03-en.pdf

3 https://sustainabledevelopment.un.org/content/documents/21252030%20Agenda%20for%20Sustainable%20Development%20web.pdf

4 https://www.integratedreporting.org/resource/international-ir-framework/

5 https://capitalscoalition.org/wp-content/uploads/2021/01/NCC_Protocol.pdf

6 https://www.oecd.org/greengrowth/

7 https://www.unep.org/explore-topics/green-economy

8 https://greeneconomytracker.org/policies/green-covid-19-recovery

9 https://sustainabledevelopment.un.org/content/documents/Agenda21.pdf

10 https://unstats.un.org/unsd/nationalaccount/sna.asp

11 https://www.wavespartnership.org/en

12 https://seea.un.org/home/Natural-Capital-Accounting-Project

13 https://aries.integratedmodelling.org/aries-for-seea-explorer/

14 https://www.eo4ea.org/

15 https://naturalcapitalproject.stanford.edu/software/invest

16 https://www.cbd.int/doc/c/abb5/591f/2e46096d3f0330b08ce87a45/wg2020-03-03-en.pdf

17 https://www.cbd.int/doc/c/cb2d/a669/83a9d4a827918b488ae8057f/sbi-03-13-add1-en.pdf

18 https://unfccc.int/sites/default/files/resource/cma3_auv_2_cover%2520decision.pdf

19 https://sustainabledevelopment.un.org/sdgs

20 https://www.un.org/millenniumgoals/

21 https://www.un.org/ga/search/view_doc.asp?symbol=A/69/L.85&Lang=E

22 https://djpr.vic.gov.au/forestry/forestry-plan

23 https://www.nasdaq.com/solutions/nasdaq-sustainable-bond-network-investors

24 https://www.climatebonds.net/2021/10/1trillion-annual-green-bond-milestone-tipped-end-2022-latest-survey-sean-kidney-calls

25 https://www.wavespartnership.org/en/policy-forum-natural-capital-accounting-better-decision-making
